# Psychological symptoms among hospital nurses in Taiwan: a cross sectional study

**DOI:** 10.1186/s12905-017-0460-5

**Published:** 2017-11-07

**Authors:** Mei-Ju Chen, Shiue-Shan Weng

**Affiliations:** 1 Department of Family Medicine, Taipei City Hospital, Heping Fuyou Branch, No.33, Sec. 2, Zhonghua Rd., Zhongzheng Dist, Taipei City, 10065 Taiwan; 2 College of healthcare administration and management, National Taipei University of Nursing and Health Science, No. 365, Mingde Rd., Beitou Dist, Taipei City, 11219 Taiwan; 3 Department of Nursing, Taipei City Hospital, Heping Fuyou Branch, No.33, Sec. 2, Zhonghua Rd., Zhongzheng Dist, Taipei City, 10065 Taiwan; 4 Institute of Public Health, National Yang-Ming University, No.155, Sec. 2, Linong St., Beitou District, Taipei City, 11266 Taiwan

**Keywords:** BSRS-5, Psychological symptoms, Self-rated health, Nurses, Low back pain

## Abstract

**Background:**

A considerable number of studies have identified the risk factors attributable to job-related stress among nurses. However, studies investigating psychological symptoms among hospital nurses is still lacking, especially in Taiwan, where the average patient to nurse ratio is among the highest in the world. This study aimed to investigate the potential role of self-rated health status, self-reported diseases, and utilization of occupational health checks in psychological symptoms among Taiwanese nurses.

**Methods:**

A cross-sectional design was conducted from September through December 2013. Data were collected through online self-administered questionnaire among 697 registered nurses in seven regional hospitals governed by the Taipei City Government.

**Results:**

Nurses with fair or poor self-rated health, lower education, <30 years of age, and low back pain were more likely to suffer from psychological symptoms. A trend toward significance was also noticed for those aged 30–39. Importantly, low back pain was the most common disease among nurses in self-reported diseases and half of the nurses reported not utilizing the occupational health examination for the last 5 years.

**Conclusions:**

To alleviate or prevent the psychological symptoms, psychosocial support, and awareness program on prevention of occupational injuries should be offered to nurses younger than 39 years old and having lower educational levels. Moreover, underutilization of occupational health examination among nurses deserves more attention.

**Electronic supplementary material:**

The online version of this article (doi: 10.1186/s12905-017-0460-5) contains supplementary material, which is available to authorized users.

## Background

Psychological symptoms have been a worldwide occupational health problem among nurses, which may adversely affect physical health, work performance, patient safety and quality of care, as well as increase costs [1–4]. In western countries, 18%–56% of the nurses were found to have depressive symptoms [5, 6]. In Asia, 38%–65% of the nurses were reported to have depressive symptoms or anxiety [1, 4, 7, 8].

The National Health Insurance Scheme (NHIS) in Taiwan is a global budget based health care insurance. The system has contributed to the recruitment of less costly nursing staff or reduction of nurse staffing levels [9]. In Taiwan, the average patient to nurse ratio in wards is 1:5–20, which is one of the highest in the world [10]. Recent surveys indicated that 40% of the nurses in Taiwan felt their workload were heavier than 3 years ago [11] and that the nursing practice rate (59.29%) was much lower than other countries [10]. Moreover, only 33% Taiwanese nurses were very likely to remain in the profession [12]. These findings highlighted the crisis of nursing shortage and poor workplace conditions in Taiwan. Studies suggest that maintaining mental wellbeing among the nurses is one of the best administration strategies to increase retention and job satisfaction [1, 7, 13]. The detection of psychological symptoms in Taiwanese nurses, therefore, is vital for further strategic and individual intervention as well as greater performance.

Self-rated health has been used as a health indicator for all dimensions of health. Many studies have shown that self- rated health or self- perceived health status is related to job stress in nurses [14, 15]. However, only limited studies have examined the association between self-rated health and psychological symptoms among nurses, except one in Lithuania [16] and the other in Taiwan [15].

In Taiwan, the Occupational Safety and Health Act mandates that the employees receive various free health check-ups in accordance to their age, gender and scope of work. A previous study showed that the utilization of Pap smear screening is related to job stress among Taiwanese nurses [17]. Yet, little is known about the relationship between utilization of health check-ups and psychological symptoms.

Psychological and musculoskeletal disorders (MSDs) are the main causes of work related sick leave and disability [18]. It has been established that psychological distress, job stress/job strain and psychosocial factors are associated with musculoskeletal complaints [19–22]. A nationwide study in Taiwan showed that the annual incidences of musculoskeletal disorders and risk of MSDs among nurses were higher than non-nurses [23]. Thus, MSDs should be taken into account when exploring factors impacting nurses’ psychological symptoms.

A considerable number of studies have identified the risk factors attributable to job-related stress among nurses. However, there is still a lack of studies investigating the factors attributable to the psychological symptoms among hospital nurses, especially in Taiwan. To fill the gap, we aimed to examine the potential role of self-rated health (SRH) status, self-reported diseases, and utilization of occupational health check-ups in psychological symptoms among Taiwanese nurses.

## Methods

### Design and sample

We adopted a cross-sectional design in this study and data were collected from self-administered internet questionnaire from September through December 2013. An additional file showed questionnaire in more detail (see Additional file 1 for self-administered questionnaire). This study protocol was approved by the institutional review board at the Taipei City Hospital (TCHIRB-1020814-E). We contacted 2300 registered nurses who worked in seven branches of the Taipei City Government and met the inclusion criterion, 697 completed the questionnaire, achieving a response rate of 30.3%.

### Instruments

#### Demographics

Demographic data collected in this study included age, gender, educational level, marital status, self-reported diseases and utilization of occupational health examination within the last 5 years.

#### Self-reported diseases

The multiple-choice question about self-reported diseases was in accordance with the Occupational Safety and Health Administration list of Current Diseases and Symptoms in a general labor health examination record, with 45 items in total (see Additional file 1 for self-administered questionnaire) [24]. Dichotomous choice was used for each disease listed.

#### Self-rated health

Self-rated health (SRH) was measured using a single-item question: “In general, how is your health?” A previous study in Taiwan had confirmed the multi-dimensional property of the single-item measure of self-rated health [25]. Respondents rated on a 5-point Likert scale to differentiate between “excellent”, “very good”, “good”, “fair” and “poor”. Because only a small number of nurses were of excellent and very good, they were merged and labeled as good.

#### Psychological symptoms

We used the 5-item brief symptom rating scale (BSRS-5) derived from the 50-item brief symptom rating scale [26] to investigate the psychological symptoms among nurses. The BSRS-5 measures the extent to which the respondents feel tense, irritated, inferior, depressed, and have trouble falling asleep in the past 7 days. The score of each item ranges from 0 (not at all) to 4 (extremely) and the summed up total score ranges from 0 to 20. The recommended cutoff point of greater than or equal to 6 was used to detect probable cases with psychological symptoms. This cutoff yielded a sensitivity of 82.6%, and specificity of 81.8% in a previous study [27].

### Statistical analysis

Statistical analysis was performed using the SPSS for Windows version 21.0. Descriptive analyses were conducted using frequency, percentage, mean, and standard deviation. Comparison between psychological symptoms and no psychological symptoms were made by chi-square test or independent t-test. Multiple logistic regression analyses were conducted to examine the relative contribution of the factors associated with psychological symptoms (BSRS-5≧6). Those factors related to psychological symptoms with two-sided *p* value ≤0.25 in the bivariate analyses were selected as candidate variables for multiple logistic regression modeling [28]. The Hosmer-Lemeshow goodness of fit test was performed to assess fitness of the models.

## Results

The characteristics of the nurses are summarized in Table 1. The average age of the nurses was 39.0 (SD = 9.4) years. Most of the nurses (89.7%, *n* = 625) completed vocational school/university education. More than half were married. The most frequently reported self-rated health status was good (76.2%) and 36.4% (*n* = 254) reported having at least one of the listed diseases, and low back pain (51.18%) was the most frequently reported (Table 2). About half (51.5%, *n* = 359) of the nurses did not receive occupational health check-ups for the last 5 years and 34.9% (*n* = 243) of them showed psychological symptoms as determined by the BSRS-5 (score of ≧6).

**Table 1:** Characteristics of Study Sample (*n* = 697)

Characteristics	Mean (SD) or n (%)	
Age, years	39.0	(9.41)
Age, years		
< 30	132	(18.9)
30–39	248	(35.6)
40–49	210	(30.1)
≧50	107	(15.4)
Educational level		
High school or lower	33	(4.7)
Vocational school/university	625	(89.7)
Postgraduate	39	(5.6)
Marital status		
Married	359	(51.5)
Divorced or widowed	14	(2.0)
Single	324	(46.5)
Self-rated health		
Poor or Fair	166	(23.8)
Good	531	(76.2)
Self-reported diseases		
No	443	(63.6)
Yes	254	(36.4)
Utilization of occupational examination within 5 years		
No	359	(51.5)
Yes	338	(48.5)
BSRS-5 score		
< 6	454	(65.1)
≧6	243	(34.9)

*Note*: *BSRS-5* 5-item brief symptom rating scale

**Table 2:** Description of Self-Reported Diseases in Taiwanese Nurses (*n* = 254)

	Number	Percent
Low back pain	130	51.18
Insomnia	41	16.14
Dermatologic disease	29	11.42
Hypertension	26	10.24
Gastrointestinal diseases	23	9.06
Urinary tract diseases	12	4.72
Thyroid gland	12	4.72
Osteoporosis	10	3.94
Diabetes mellitus	9	3.54
Others	89	35.04

As shown in Table 3, nurses who were younger and with poorer self-rated health, self-reported diseases, and low back pain were more likely to suffer from psychological symptoms. No significant association was found between the utilization of occupational health check-ups for the last 5 years and psychological symptoms.

**Table 3:** Comparison between Characteristics and Psychological Symptoms in Taiwanese Nurses (*n* = 697)

	Without psychological symptoms (*n* = 454)		With psychological symptoms (*n* = 243)		*p* value
	*n*	%	*n*	%	
Age, years					0.003
< 30	77	17.0	55	22.6	
30–39	148	32.6	100	41.2	
40–49	155	34.1	55	22.6	
≧50	74	16.3	33	13.6	
Educational level					0.071
High school or lower	22	4.8	11	4.5	
Vocational school/university	400	88.1	225	92.6	
Postgraduate	32	7.0	7	2.9	
Marital status					0.808
Married	236	52.0	123	50.6	
Divorced or widowed	10	2.2	4	1.6	
Single	208	45.8	116	47.7	
Self-rated health					<0.001
Poor or Fair	68	15.0	98	40.3	
Good	386	85.0	145	59.7	
Self-reported diseases					<0.001
No	312	68.7	131	53.9	
Yes	142	31.3	112	46.1	
Low back pain					
No	388	85.5	179	73.7	<0.001
Yes	66	14.5	64	26.3	
The utilization of occupational examination for the last 5 years					0.160
No	225	49.6	134	55.1	
Yes	229	50.4	109	44.9	

*Note*: *S.D.* standard deviation

The multiple logistic regression results showed that age, educational level, self-rated health, and low back pain were significantly associated with psychological symptoms. Nurses younger than 30 years of age were more likely to suffer from psychological symptoms compared with those aged ≧50 (OR = 2.00; *p* = 0.026); there was a marginal higher level of psychological symptoms for those aged 30–39 (OR = 1.72; *p* = 0.055); no significant difference was found in the 40–49 age groups. Nurses with an educational level of high school or lower (OR = 4.64; *p* = 0.012) or vocational school/university (OR = 2.85; *p* = 0.021) were more likely to suffer from psychological symptoms than those who with postgraduate degree. Fair or poor self-rated health was associated with an increased risk of psychological symptoms (OR = 3.67; *p* < 0.001). Those who had low back pain were more likely to suffer from psychological symptoms (OR = 1.56; *p* = 0.042) (Table 4).

**Table 4:** Multiple Logistic Regression Model for Factors Associated with Psychological Symptoms in Taiwanese Nurses (*n* = 697)

Variable	B	S.E.	Wald	OR	95%CI	*p* value^a^
Age (years)						
< 30	0.69	0.31	4.98	2.00	1.09–3.67	0.026
30–39	0.54	0.28	3.67	1.72	0.99–3.00	0.055
40–49	−0.03	0.29	0.01	0.97	0.55–1.71	0.914
≧50				1		
Educational level						
High school or lower	1.53	0.61	6.36	4.64	1.41–15.28	0.012
Vocational school/university	1.05	0.45	5.32	2.85	1.17–6.92	0.021
Postgraduate				1		
Self-rated health						
Fair or poor	1.30	0.20	42.521	3.67	2.48–5.43	<0.001
Good				1		
Low back pain						
No				1		
Yes	0.44	0.22	4.13	1.56	1.02–2.39	0.042

*Note: S.E.* standard error, *OR* odds ratio, *CI* confidence interval

^a^ Variables with a *p* value of greater than .05 were excluded from the model

## Discussion

Recent studies on psychological symptoms among nurses mostly focused on factors related to workplace stress and burnout. Both workplace stress and burnout are positively associated with symptomatology and depression [15, 29]. Our study demonstrated that psychological symptoms were associated with fair or poor self-rated health, low education, age below 30, and low back pain among nurses. Although they have better knowledge about health, only 48.5% of them used occupational health checkups for the last 5 years (48.5%) and no association was found between occupational health check-ups and psychological symptoms. To date, only one study has addressed the association between psychology and health checkups and concluded that stress was in relation to the use of Pap smear [17]. The levels of perceived stress may provoke the people to accept the disease screening [30]. Further investigation on underlying reasons for lower use of occupational health check-ups and the association between psychological symptoms and particular health checkups items are warranted.

The present study found that nurses with fair or poor self-rated health were more likely to suffer from psychological symptoms, suggesting that self-rated health may be used as an indicator of psychological symptoms in nurses. Existing literature indicates that shift work schedules, sleep quality and long working hours may contribute to nurses’ poor health status [14, 31]. It is important to provide flexible shift work schedules, reasonable work hours and psychosocial resources for nurses in order to improve their health and emotional well-being.

Consistent with existing literature, our bivariate analysis revealed that self-reported diseases were correlated with psychological symptoms [1, 5]. However, this variable was excluded from the model after adjusting for low back pain. Given that context, low back pain may be a predominant predictor for psychological symptoms in comparison to other self-reported diseases. In accord with other studies [32], our findings showed that low back pain was the most common disease among nurses. Nursing tasks involving prolonged standing and patients lifting may be one of the causes for low back pain. The high incidence of low back pain among nurses may imply not only incorrect work-related posture but also high job stress and insufficient rest [19, 21]. One systematic review and meta-analysis showed that low work-related social support is related to increased incidence of back pain [21]. Work-related social support and education on prevention for occupational injuries, thus, should be taken into account to reduce low back pain incidents and psychological symptoms.

Younger nurses have less work experiences, longer rotating night shifts and spend more time to be familiar with their work as compared with older nurses [13, 33], resulting in a higher risk for work-related stress and burnout [29, 34]. A previous study also showed that mid-age nurses were in lower levels of stress, emotional exhaustion and depression compared to younger nurses and suggested that more productive responses to the role stressors will be developed once they become mid-aged [35]. Our findings showed that being younger than 30 years was significantly related to psychological symptoms and there was also a trend for those aged 30–39 that should be noticed. These highlight the need to offer psychosocial support and develop stress awareness and management program for young nurses, especially for those who were younger than 30 years old. Educational level may have a protective effect on emotional well-being [36], however this is seldom considered in nurses’ health studies. A previous study explained that taking a higher education is related to a better socioeconomic level, and might resulting in experiencing lower strain and hardship [37]. In our study, we found that, in the strain of workplace, nurses with lower educational levels were more likely to suffer from psychological symptoms. The possible explanation is that such mental resources give higher resilience in regards to strain or stress and protect against psychological symptoms. In 2009, an Australian study [38] showed that working long hours, enduring psychological stress and coping with high client expectations, performing euthanasia, experiencing compassion fatigue and burnout are all factors that increase the risk of suicide for some professions. Our findings suggested that hospital managers should establish further intervention program for promoting mental health. Mental health in the workplace is a complex issue for nurses and the managers. Knowledge about the effectiveness of interventions is indispensable to changing these figures and improving nurses’ mental health.

## Limitations

Our study is with limitations. First, the response rate of this study was low (30.3%). The non-response bias could be attributed to unwillingness to participate, fear of disclosure of psychological weakness, heavy workload and lack of incentives. In addition, due to the use of a cross-sectional design, a causal relationship could not be established. Another limitation is that we only used BSRS-5 and did not combine other measurements or clinical diagnosis for comparison of psychological symptoms. Nevertheless, the 5-items questionnaire is easy to use and suitable for the evaluation of psychological symptoms in workplace [39].

## Conclusions

Psychological symptoms were associated with fair or poor self-rated health, low education, <30 years of age, and low back pain among nurses. We recommend psychosocial support and stress awareness and management program for nurses susceptible to the development of occupational psychological symptoms. Also, reasons causing the low intention to use occupational health checkups should be reviewed for correction of the deficiency.

## Additional file

Additional file 1: Self-administered questionnaire. This is the self-administered questionnaire used in the study. (DOCX 15 kb)

## Abbreviations

BSRS-5: 5-item brief symptom rating scale
NHIS: National Health Insurance Scheme
