# Supplementary material for: Psychological symptoms among hospital nurses in Taiwan: a cross sectional study
Source: BMC Womens Health. 2017 Nov 7;17:101. doi: 10.1186/s12905-017-0460-5 (PMC5678555; doi:10.1186/s12905-017-0460-5)
Supplement: Additional file 1: — Self-administered questionnaire. This is the self-administered questionnaire used in the study. (DOCX 15 kb) [file 12905_2017_460_MOESM1_ESM.docx]

**Self-administered questionnaire**

1. **Demographics**

| 1. | Gender | (1)□Male　(2)□Female |
| --- | --- | --- |
| 2. | Birthday | A.D. |
| 3. | Educational level | (1)□Elementary school (2)□Junior high school  (3)□High school (4)□ Vocational school  (5)□university (6)□ Postgraduate |
| 4. | Marital status | (1)□Single　(2)□Married　(3)□Widowed  (4)□Divorced |

1. **Health Status**

| 1. | In general, how is your health? | | (1)□Excellent　(2)□Very good　(3)□Good (4)□Fair (5)□Poor | | | | |
| --- | --- | --- | --- | --- | --- | --- | --- |
| 2. | Do you have any current diseases or symptoms? | | | | | | |
|  | (1)□No  (2)□Yes( Please choose the following multiple-choice) | | | | | | |
|  | current diseases:  (1)□hypertension (2)□diabetes mellitus (3)□heart disease (4)□cancer  (5)□cataract (6)□cerebral vascular accident (7)□ seizure (8)□asthma (9)□chronic bronchitis/ emphysema (10)□tuberculosis (11)□kidney disease (12)□liver disease (13)□anemia (14)□otitis media  (15)□hearing impairment (16)□thyroid disease  (17)□peptic ulcer/ gastritis (18)□gastroesophageal reflux disease  (19)□ fracture (20)□surgery (21)□other chronic disease | | | | | | |
|  | current symptoms:  (1)□cough (2)□expectoration (3)□dyspnea (4)□chest pain  (5)□palpitations (6)□vertigo (7)□headache (8)□tinnitus  (9)□fatigue (10)□nausea (11)□abdominal pain  (12)□constipation (13)□diarrhea (14)□hematochezia  (15)□upper back pain (16)□low back pain (17)□limb numbness  (18)□joint pain (19)□dysuria (20)□frequent urination  (21)□muscle weakness (22)□weight loss ≥3 kg (23)□other symptom | | | | | | |
| 3. | Please answer the following perceived level of psychological distress in the past 7 days. | | | | | | |
|  |  | Not at all  (0) | | a little bit  (1) | moderately  (2) | quite a bit  (3) | Extremely  (4) |
| (1) | feeling tense or keyed up(anxiety) |  | |  |  |  |  |
| (2) | feeling low in mood (depression) |  | |  |  |  |  |
| (3) | feeling easily annoyed or irritated (hostility) |  | |  |  |  |  |
| (4) | feeling inferior to others (interpersonal hypersensitivity: inferiority) |  | |  |  |  |  |
| (5) | having trouble falling asleep (insomnia) |  | |  |  |  |  |

1. **The Utilization of Health Examination**

| 1. | Do you have occupational examination in past 5 years? | |
| --- | --- | --- |
|  | (1)□No  (2)□Yes |  |
